# Development of a Point-of-Care SPR Sensor for the Diagnosis of Acute Myocardial Infarction

**DOI:** 10.3390/bios13020229

**Published:** 2023-02-05

**Authors:** Sunil Choudhary, Zeynep Altintas

**Affiliations:** 1 Institute of Chemistry, Faculty of Maths and Natural Sciences, Technical University of Berlin, Straße des 17. Juni 124, 10623 Berlin, Germany; 2 Institute of Materials Science, Faculty of Engineering, Kiel University, 24143 Kiel, Germany; 3 Kiel Nano, Surface and Interface Science (KiNSIS), Kiel University, 24118 Kiel, Germany

**Keywords:** SPR, point-of-care sensor, epitope-imprinting, nanoMIPs, cTnI, myocardial infarction

## Abstract

A novel point-of-care surface plasmon resonance (SPR) sensor was developed for the sensitive and real-time detection of cardiac troponin I (cTnI) using epitope-imprinted molecular receptors. The surface coverage of a nano-molecularly imprinted polymer (nanoMIP)-functionalized SPR sensor chip and the size of nanoMIPs (155.7 nm) were characterized using fluorescence microscopy and dynamic light scattering techniques, respectively. Atomic force microscopy, electrochemical impedance spectroscopy, square wave voltammetry and cyclic voltammetry techniques confirmed the successful implementation of each step of the sensor fabrication. The SPR bio-detection assay was initially established by targeting the cTnI peptide template, and the sensor allowed the detection of the peptide in the concentration range of 100–1000 nM with a correlation coefficient (R^2^) of 0.96 and limit of detection (LOD) of 76.47 nM. The optimum assay conditions for protein recognition were subsequently determined, and the cTnI biomarker could be detected in a wide concentration range (0.78–50 ng mL^−1^) with high reproducibility (R^2^ = 0.91) and sensitivity (LOD: 0.52 ng mL^−1^). The overall sensor results were subjected to three binding isotherm models, where nanoMIP-cTnI interaction followed the Langmuir binding isotherm with the dissociation constant of 2.99 × 10^−11^ M, indicating a very strong affinity between the cTnI biomarker and epitope-imprinted synthetic receptor. Furthermore, the selectivity of the sensor was confirmed through studying with a control nanoMIP that was prepared by imprinting a non-specific peptide template. Based on the cross-reactivity tests with non-specific molecules (i.e., glucose, p53 protein, transferrin and bovine serum albumin), the nanoMIP-SPR sensor is highly specific for the target biomarker. The developed biomimetic sensor, relying on the direct assay strategy, holds great potential not only for the early and point-of-care testing of acute myocardial infarction but also for other life-threatening diseases that can be diagnosed by determining the elevated levels of certain biomarkers.

## 1. Introduction

Cardiovascular disease (CVD) is a term which is related to the disorders associated with the heart and blood vessels. It usually occurs due to the deposition of fat in the arteries and an increased risk of blood clots [1]. Being the leading cause of increasing fatality rates worldwide, CVDs have been viewed as one of the major problems not only in the wealthy but also in industrialized nations for decades, and the need for rapid and easy detection with timely diagnosis has the utmost importance [2]. The World Health Organization (WHO) estimates that 17.9 million people worldwide die of cardiovascular disease each year, accounting for 32% of all deaths and significant morbidity. Of these deaths, 85% are attributable to strokes and heart attacks, which have increased by 5% over the previous five years [3]. Patients who are at high risk of developing CVD may have some of the symptoms that can be monitored in a medical institution, such as high blood pressure, elevated cholesterol levels and obesity. The chances of efficient treatment and higher survival rates have been hindered by delayed diagnosis. Therefore, identifying those patients who are at high risk of CVDs at the initial stage and providing them with appropriate treatment helps to prevent significant premature deaths. This may also help in cutting the cost by screening the hospital admission processes and focusing on the resources for those that are specifically at risk. In this regard, biomarkers and biosensors play a vital role to revolutionize the diagnosis of CVD [3,4].

The detection of CVDs can be achieved by determining the concentration of certain substances known as biomarkers which are specific to the respective diseases. Hence, diagnostic and prognostic biomarkers are required to be detected with minimally invasive methodologies to improve the management of CVDs. These biomarkers can also help to detect the severity of a particular cardiac disorder [5,6,7]. Extensive efforts are necessary to explore and develop highly efficient, rapid, accurate, easy-to-use, sensitive and reliable sensing devices for the detection of CVDs.

As troponin is a regulatory protein that regulates muscle contractions and is elevated above normal levels for 4 to 10 days after symptoms eventuate, it is beneficial for detecting individuals with late-stage acute myocardial infarction (AMI) [8]. Human troponin is made up of three subunits: TnC, TnT and TnI. The latter is used as a gold standard marker because of its exceptional specificity and sensitivity for the detection of AMI, as well as the fact that it is found in heart muscles when there is a myocardial cell injury and has a higher blood concentration [9]. The level of cTnI drastically increases in the bloodstream from the threshold level of 0.04 ng mL^−1^ to 1.4 ng mL^−1^ within 3–12 h [10].

cTnI detection utilizing biosensor technology has been the subject of numerous studies in the past and continues to grow. For instance, Mason et al. developed a sensitive and real-time fiber-optic-based SPR sensor for cTnI that could successfully detect the biomarker down to 3 ng mL^−1^ within 10 min [11]. Fan et al. developed a novel label-free photoelectrochemical (PEC) sensor allowing the detection of cTnI in the concentration range of 0.001–50 ng mL^−1^ with a limit of detection (LOD) of 0.3 pg mL^−1^, good stability and reproducibility [12]. In other work, Wu et al. have reported on an ultrasensitive immunoassay for cTnI quantification based on magnetic field-assisted SPR, revealing an LOD of 1.3 ng nL^−1^ [13]. Mansuriya and Altintas have developed an enzyme-free nano-immunosensor for cTnI detection using gold nanoparticles and graphene quantum dots as enzyme mimics and signal amplification agents, where the disposable electrochemical sensor could achieve the determination of the biomarker in a wide concentration range (10–1000 pg mL^−1^) with an LOD of 0.5 pg mL^−1^ in 50% diluted human serum [14]. 

All of the aforementioned works used antibody-based receptors, which are bearing fragile and costly biosensing platforms. Moreover, the sensor development process and/or the instrumentation in these works are laborious. Thanks to the continuous developments in the molecular imprinting field, some elegant sensor approaches using MIP-based synthetic receptors have recently been introduced for the detection of cardiac biomarkers. Baldoneschi et al. reported on cTnI detection using a poly-norepinephrine based optical MIP sensor, which was prepared by drop casting of the polymer films on SPR chip sensors. The study uses two alternative peptide sequences (aar C-terminal 197–210: ALSGMEGRKKKFES and N-terminal 28–40: 28AYATEPHAKKKSK) as templates in the epitope imprinting process. The sensor offers high affinity (K_D_ = 4.4 nM) and selectivity yet requires bulky instrumentation (Biacore, GE Healthcare) and a sandwich assay by utilizing a detector antibody to achieve enough sensitivity [15]. 

McClements et al. recently developed a thermal assay for the detection of cTnI using epitope-mediated nanoMIPs on screen-printed electrodes. The disposable sensor was coupled with three different nanoMIP immobilization approaches (i.e., drop casting, dip coating and a covalent approach relying on electrografting with an organic coupling reaction), where the covalent functionalization of nanoMIPs onto the surface provided the most reproducible sensing results. Through monitoring changes in heat transfer at the solid−liquid interface, the authors could measure concentrations as low as 10 pg L^−1^ in buffer. This novel technique might be suitable for commercialization purposes; however, it currently requires a much longer assay time (45 min PBS injection to obtain a baseline followed by 12 min injection of sample and another 30 min to stabilize the system for the next injection) [16] than that of the current work. In another work, the same research group coupled their thermal detection approach with nanoMIP-based multiplex sensor platform for the quantification of other cardiac biomarkers (heart-fatty acid binding protein and ST2) and demonstrated that the system could selectively detect both cardiac biomarkers within the physiologically relevant range with LODs of 4.18 and 8.79 ng mL^−1^, respectively. Nevertheless, the use of a whole protein imprinting approach in this work makes it high-cost [17].

MIPs were also introduced to the femtomole detection of cTnI peptides using matrix-assisted laser desorption ionization mass spectrometry (MALDI-TOF-MS) [18]. The synthetic receptors provided a moderate selectivity with imprinting factors ranging from 3 to 5 with dissociation constants of 58–817 nM. However, the complicated assay procedure, as well as the laborious instrumentation, are not suitable for providing a portable sensing approach that can be used in point-of-care testing.

In the present work, nanoMIPs were used as synthetic receptors to reduce the overall cost and provide a label-free and direct assay strategy with a portable and user-friendly device for the highly sensitive detection of cTnI. The receptors were manufactured using a partial imprinting approach [19,20,21], relying on the molecular imprinting of a cTnI-derived small peptide (aar 40–49: ISASRKLQLK) immobilized on a solid phase prior to polymerization [22,23,24,25,26,27,28,29]. The resulting nanoMIPs were characterized using dynamic light scattering (DLS) and fluorescence microscopy techniques. The sensor assays were successfully established by initially targeting cTnI-derived peptides, followed by protein detection studies in wide concentration ranges. All steps of sensor fabrication were characterized by employing cyclic voltammetry (CV), square wave voltammetry (SWV), electrochemical impedance spectroscopy (EIS) and atomic force microscopy (AFM) techniques. The sensitivity, affinity, selectivity and specificity of the sensor were successfully confirmed towards developing the first nanoMIP-SPR sensor for cTnI detection using epitope-imprinted synthetic receptors and a label-free bioassay.

## 2. Materials and Methods

### 2.1. Materials and Reagents

First, 11-mercaptoundecanoic acid (MUDA), N′-tetramethylethylenediamine (TEMED), 1-ethyl-3-(3-dimethylaminopropyl) carbodiimide (EDC), glutaraldehyde (GA), phosphate-buffered saline (PBS), sodium acetate, ethanol, acetone, N-isopropylacrylamide (NIPAm), N,N′ methylenebisacrylamide (BIS), acrylic acid (AAc), N-(3-aminopropyl) methacrylamide hydrochloride (APM), N-hydroxysuccinimide (NHS), 3-aminopropyltriethoxysilane 3- (APTES), ethanolamine, methacryloxy ethylthiocarbamoyl rhodamine B, N-tert-butylacrylamine (TBAm), ammonium persulphate (APS), Tween 20 (polyoxyethylenesorbitan monolaurate), sodium borohydride and anhydrous toluene were purchased from Sigma Aldrich (Steinheim, Germany). In order to obtain double-distilled water, a Millipore Direct-Q 3 UV (Millipore, Taufkirchen, Germany) was used. The glass beads (∅100 μm) were bought from Carl Roth (Karlsruhe, Germany). Carl Roth also provided the syringe filters (0.45 and 0.22 m Rotilabo PTFE) (Karlsruhe, Germany). Without additional purification, analytical or HPLC grade chemicals and solvents were employed throughout. A 0.22 m syringe filter was used to filter a PBS/Tween (phosphate buffered saline +0.05% Tween) buffer.

### 2.2. Apparatus and Equipment

Using DLS (Zetasizer pro, Malvern, UK), the size of nanoMIPs was determined. Fluorescence microscopy photos were taken by employing the Keyence BZ-X800 (Osaka, Japan) series to show the uniform distribution and successful immobilization of nanoMIPs on the sensor surface. A PalmSens4 compact electrochemical interface with a three-electrode system (PalmSens4 workstation, Beltec, Germany) was employed for the electrochemical characterization of the sensor preparation. The alterations in the sensor surface from the bare gold chip to the immobilized nanoMIP and protein capture were also verified using the AFM NanoWizard II (JPK Instruments AG., Berlin, Germany). The gold-coated sensor chips (12.4 × 7 × 0.7 mm) for SPR tests were purchased from Plasmetrix (Montreal, QC, Canada). Bio-detection assays were performed using a portable SPR device (CORGI IIF, Plasmetrix). The apparatus included two flow channels to accommodate target and control assays. A peristaltic pump (Ismatec Reglo ICC, Digital pump, 2-channel, Cole-Parmer, Wertheim, Germany) and microtubing setup were also added to inject the samples in a continuous fluid flow. The channel was divided on the chip’s surface using a silicon gasket. Further details about the device can be found on the website of the provider (https://plasmetrix.com/products/ (15 January 2023)).

### 2.3. Reagent Preparation

Two PBS tablets were dissolved in 400 mL of double-distilled water at room temperature to create a 0.01 M PBS buffer solution (pH 7.4). It was kept at 4 °C when not in use and employed for the solid-phase synthesis of nanoMIPs. A 0.05% *v*/*v* solution of Tween20 (Sigma Aldrich, Berlin, Germany) was dissolved in the PBS solution to obtain PBS/Tween (PBS/T) buffer to be used in biosensor assays throughout the study. This buffer was filtered through a 0.22 m syringe filter, degassed at ambient temperature in a vacuum and then kept at 4 °C until it was needed.

Aqueous redox marker solution comprising K_3_[Fe(CN)_6_] and KCl was prepared to be used in all electrochemical measurements for sensor surface characterization. The redox marker solution (10 mM K_3_[Fe(CN)]_6_ with 0.1 M KCl) was freshly prepared for each experiment. To prevent photodegradation, the solution was completely blended and covered with aluminum foil after preparation.

### 2.4. Preparation of Epitope-Immobilized Microbeads for NanoMIP Synthesis 

**Scheme 1:**
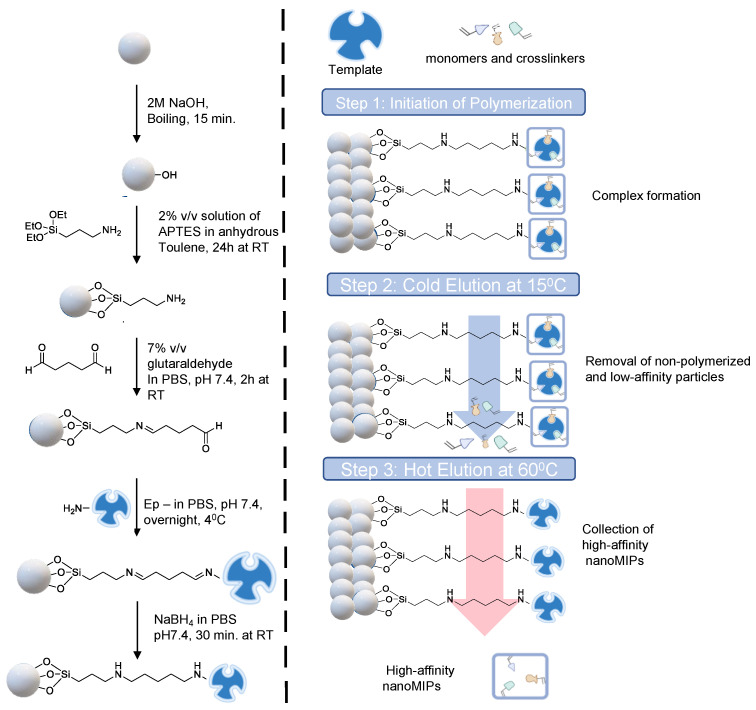
Schematic representation of epitope-template immobilization on glass beads as solid support (**left**) and three-step solid-phase synthesis (**right**).

Glass beads (60 g) were activated by boiling in NaOH (2 M, 40 mL) for 15 min. The beads were then washed with double distilled water until they reached a pH of 7.4 before being fully dried with nitrogen. The beads were then salinized by incubating them for 24 h at room temperature in an inert atmosphere in a solution of 2% *v*/*v* APTES diluted in 24 mL of anhydrous toluene. Afterwards, the beads were rinsed several times in acetone (500 mL) and methanol (60 mL) to eliminate any remaining APTES solution before drying. To create aldehyde-functionalized glass beads, a 5% *v*/*v* solution of glutaraldehyde dissolved in PBS (24 mL) was incubated for 2 h at room temperature (Scheme 1).

Further, the beads were washed with double-distilled water (480 mL) under vacuum using the Buchner funnel and flask. The epitope template (7 mg) was then dissolved in PBS (40 mL) and added to the glass beads in a beaker, followed by overnight incubation at 4 °C. The unbound peptide molecules were eliminated the next day by multiple washings with double-distilled water (10 × 25 mL). The glass beads were incubated in NaBH_4_ solution in PBS (24 mL) for 30 min at room temperature in order to minimize Schiff bases. The glass beads were incubated with an ethanolamine solution (1 mM, 50 mL, diluted in PBS) for 15 min to block the peptide-free surface, avoiding non-specific associations during polymerization, after being dried and rinsed with double-distilled water after 30 min. The glass beads were then dried under a vacuum and kept in the refrigerator for nanoMIP synthesis.

### 2.5. Synthesis of NanoMIPs

The general principle of the solid phase synthesis method has been illustrated in Scheme 1. The procedure used to synthesize the cTnI-specific MIP receptors was derived from Altintas et al. [30]. The following monomers were added to 95 mL of double-distilled water in a quick manner: 39 mg of N-isopropyl acrylamide, 2 mg of N, N′-methylene bisacrylamide, and 58 mg of N-(3-aminopropyl)methacrylamide hydrochloride, each dissolved in 1 mL of double-distilled water. All of these monomers were added, and the mixture was agitated for 30 min. After stirring for 30 min, 33 mg of N-tert-butyl acrylamide, 3 mg of methacryloxyethyl and 3 mg of thiocarbamoyl rhodamine B were added. The mixture was then sonicated for 20 min. The initiator, ammonium persulfate, was added to the polymerization mixture at a dose of 48 mg. To eliminate atmospheric oxygen, the mixture was sonicated for 20 min and then purged with dry nitrogen gas for 20 min.

The polymerization was initiated by adding 24 µL of TEMED (activator) to the mixture, and it took place for 2 h at room temperature in a nitrogen environment. The polymerized mixture and the glass beads were transferred to a 60 mL solid phase extraction (SPE) cartridge after 2 h of polymerization, and the bottom entrance was sealed with a 20 µm polyethene frit to prevent leaking. By using three portions of a cold wash of double-distilled water (15 °C, 20 mL), low-affinity particles, unbounded monomers and fragments were eliminated. To obtain the high-affinity MIP nanoparticles, a hot elution was then performed with double distilled water (65 °C, 20 mL). This hot elution process was repeated 7 times, and a total volume of 140 mL nanoMIP solution was collected. The resulting solution was freeze-dried with liquid nitrogen, and the lyophilized nanoMIPs were dissolved in double distilled water to obtain a 1 mg mL^−1^ nanoMIP solution and stored at 4 °C until use.

### 2.6. DLS Size Analysis of NanoMIPs

The investigation of the size distribution of nanoMIPs was carried out utilizing DLS. The light scattering method is based on the monochromatic light scattering that occurs when it strikes a particle. This scattering is then detected by a suitable detector, which enables the determination of a variety of characteristics, such as the radius of gyration molecular weight, diffusion coefficient, etc. The nanoMIPs were dissolved in the buffer to provide a standard solution of 1 mg mL^−1^. Additionally, the solution was sonicated at 20 °C for 40 min before being filtered through a 0.22 µm syringe filter. The DLS samples were once more sonicated for 40 min in identical conditions, and then they were transferred using a syringe to the DLS vial. The Malvern zetasizer pro DLS was used to measure the size distribution after the vial was gently inserted into it. All measurements were performed at a steady temperature (25 °C).

### 2.7. NanoMIPs Immobilization on MUDA-Coated SPR Sensor Chips

The nanoMIP solution was prepared using PBS/T buffer to achieve 1 mg mL^−1^ or 750 mg mL^−1^ solution, which was homogenized and subjected to a 30 min sonication process to remove any agglomerates. After filtering the MIP solution via a 0.45 µm syringe filter, it was placed in a freezer at 4 °C until it was needed. All of the solutions (PBS/T buffer and ethanolamine) were prepared and kept at room temperature prior to the beginning of SPR assays. The gold chip was gently placed over the SPR prism of the CORGI IIF SPR System after it had been cleaned using ethanol. With the aid of a peristaltic pump, the flow rate was maintained at 4 μL min^−1^ throughout the assays. After that, PBS/T buffer was used to prime the surface across both SPR flow channels until a stable baseline was obtained. 

**Scheme 2:**
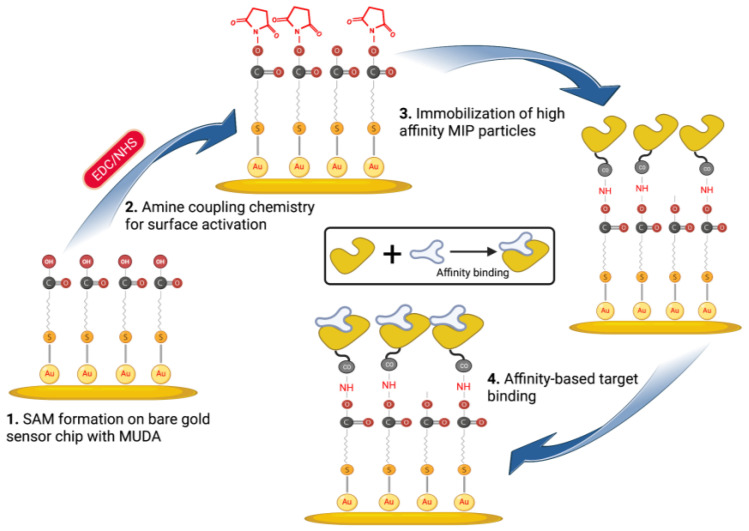
Affinity-based sensor assays for cTnI detection using epitope-imprinted nanoMIPs.

In order to activate the carboxyl groups on the sensor chip surface formed by MUDA, a 1:1 volume ratio mixture of EDC (0.4 M) and NHS (0.1 M) was dissolved in double-distilled water and injected for 4 min (Scheme 2). A 0.1 M sodium acetate buffer solution was then flushed for 2 min after the EDC/NHS coupling in order to stabilize the NHS ester. After the MIP nanoparticles (750 μg mL^−1^) were covalently immobilized for 8 min, ethanolamine (0.1 M) was injected for 4 min to block the MIP-free areas on the surface and avoid non-specific binding during detection studies. Following the addition of nanoMIPs and ethanolamine, PBS/T was flushed for 2 min.

### 2.8. Bioassays for Peptide and Protein Detection

Cumulative binding experiments were carried out for the detection of cTnI-derived peptides using the SPR sensor. For this, epitope template dissolved in PBS (pH 7.4) was injected to the nanoMIP immobilized sensor surface at increasing concentrations (1–1000 nM). The SPR response unit (RU) error for channel I was calculated using the standard deviation of the PBS control signal on channel II. To wash away the unbound peptide molecules after each sample injection for 10 min, the surface was rinsed by injecting PBS/T for 2 min. 

For protein detection assays, different concentrations of cTnI (10, 100 and 1000 ng mL^−1^) were initially tested by studying with three injection times (5, 10 and 15 min) to determine the optimum duration for efficient capture of the protein biomarker by nanoMIPs. Similar to the peptide detection assays, cTnI samples were prepared in a wide concentration range and then injected to the sensor surface for 10 min based on the optimized conditions. Between samples injections, the weakly bound or unbound protein molecules were washed away with PBS/T injection for 2 min. 

## 3. Results and Discussion 

Molecularly imprinted polymers have been extensively studied in a variety of domains as recognition elements owing to their desirable features [31,32,33,34,35]. However, there still exist major challenges in imprinting large molecules, such as proteins, due to their substantial size as well as conformational changes during the polymerization process, which leads to a plethoric heterogeneity in the formation and size of resulting cavities, as well as binding sites inside the cavities [36]. The very high cost of proteins also makes it difficult to consider them as a template as a whole for the molecular imprinting process. Hence, there has been a growing and constant interest in epitope imprinting, which appeases the aforementioned limitations to a great extent and provides high-performance biosensing platforms for protein detection [20,37]. Herein, for the first time, we brought the superior features of epitope imprinting, solid-phase synthesis, and a portable SPR together to establish an innovative and user-friendly sensing system for the detection of cTnI. 

### 3.1. NanoMIP Characterization 

**Figure 1:**
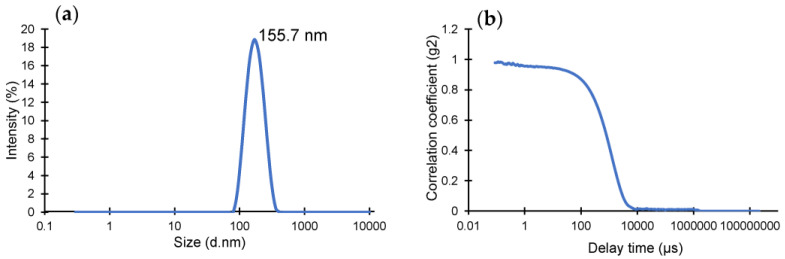
(**a**) Hydrodynamic size distribution of cTnI-specific epitope-imprinted nanoMIPs. (**b**) Correlation fit of DLS data confirming the quality of the measurement.

The yield of the lyophilized nanoMIPs was determined as 6.5 mg using a microbalance. DLS operates under the premise of determining the hydrodynamic size of the particle by detecting the Brownian motion of the particles in the solution. The homogeneity, quality and size of the produced nanoMIPs were identified using DLS (Figure 1), where the hydrodynamic radius and polydispersity index (PDI) of the particles in solution were found to be 155.7 nm and 0.1543, respectively. The very low PDI value indicates highly monodisperse and homogenous polymer particles in the solution. 

### 3.2. NanoMIP Immobilization and Target Detection Assays 

An SPR sensor (CORGI IIF, Plasmetrix) was employed to determine the sensitivity, affinity, selectivity and specificity of the epitope-mediated nanoMIPs towards the target molecules. The portable sensing platform allows the changes taking place on the chip surface in real-time to be measured. Prior to docking the sensor chip to the device, a self-assembled monolayer (SAM) was formed on its surface with the overnight incubation of MUDA (2 mM), followed by rinsing with ethanol and double-distilled water and gentle drying with a nitrogen stream. After priming the sensor with PBS/T buffer to obtain a baseline, the reactive succinimide esters were introduced to the MUDA-coated surface by activating the carboxyl groups using EDC-NHS coupling chemistry. The nanoMIPs were covalently immobilized onto the surface with the aid of NHS-ester groups. To realize the most convenient receptor immobilization conditions, two different concentrations of nanoMIPs (0.75 and 1 mg mL^−1^) were tested with two different incubation formats (i.e., 4 + 4 min incubation and 8 min incubation). The preliminary studies have suggested 0.75 mg mL^−1^ concentration and 8 min incubation time as the optimum for efficient and consistent binding results. Thus, all the further assays were carried out using these conditions. 

**Figure 2:**
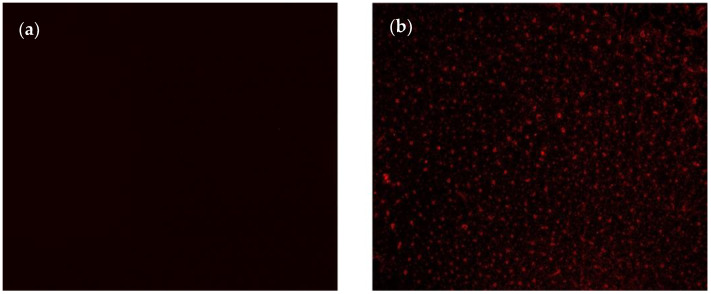
Fluorescent microscopy images of sensing surface (**a**) after MUDA coating and (**b**) nanoMIP immobilization.

The successful immobilization of nanoMIPs was confirmed using the fluorescence microscopy technique by imaging the sensor surface before and after nanoMIP immobilization (Figure 2). Thanks to the incorporation of rhodamine into the polymerization mixture during nanoMIP synthesis, the fluorescence microscope could show the uniform distribution of synthetic receptors as spherical and uniformly distributed red particles on the sensor surface (Figure 2b). 

**Figure 3:**
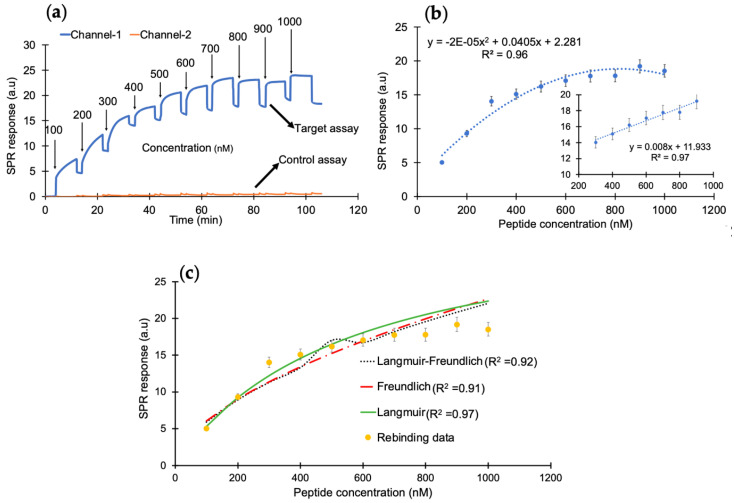
(**a**) The SPR sensorgram of peptide rebinding within a concentration range of 100–1000 nM. (**b**) The concentration-dependent peptide binding with a correlation coefficient of R^2^ (0.97) for the linear range of 300–900 ng mL^−1^. (**c**) The binding-isotherm of peptide rebinding for a concentration range of 100–1000 nM.

After successfully confirming the immobilization of nanoMIPs, the cumulative rebinding assays with cTnI-derived peptides was performed in the concentration range of 100–1000 nM (Figure 3a). Herein, channel 1 was used for the target rebinding assay, whereas channel 2 was utilized for the control assay. Figure 3b illustrates the concentration-dependent peptide binding assay in the entire range with an R^2^ value of 0.96. The changes in the sensor signal showed linearity from 300 to 900 nM with an R^2^ value of 0.97 (inset). To determine the binding affinity between the peptide and the synthetic receptors, kinetic data analysis was performed by subjecting the overall peptide detection data into binding isotherm models, where the binding interactions followed the Langmuir model with an R^2^ value of 0.97, suggesting one-to-one binding between molecular receptors and peptide molecules (Figure 3c). 

**Figure 4:**
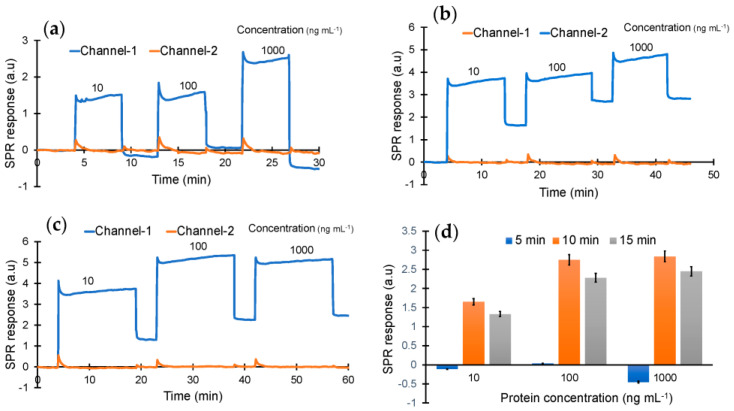
Real-time protein binding assay for the optimization of concentration (10, 100 and 1000 ng mL^−1^) and incubation time (**a**) 5 min, (**b**) 10 min and (**c**) 15 min. (**d**) Overall performance of the sensor with the different concentrations and incubation times.

As the next step, cTnI protein detection was studied using the epitope-mediated nanoMIP-SPR sensor. For this, the optimum injection time for capturing the protein target was first investigated by testing three different concentrations of cTnI (10, 100 and 1000 ng mL^−1^) with three different injection durations (5, 10 and 15 min) (Figure 4). These preliminary studies confirmed that the sensor is able to quantify the target biomarker at all three concentrations with injection times of 10 and 15 min, whereas a 5 min injection was found to be insufficient for molecular interactions between the receptor and cTnI biomarker (Figure 4).

**Figure 5:**
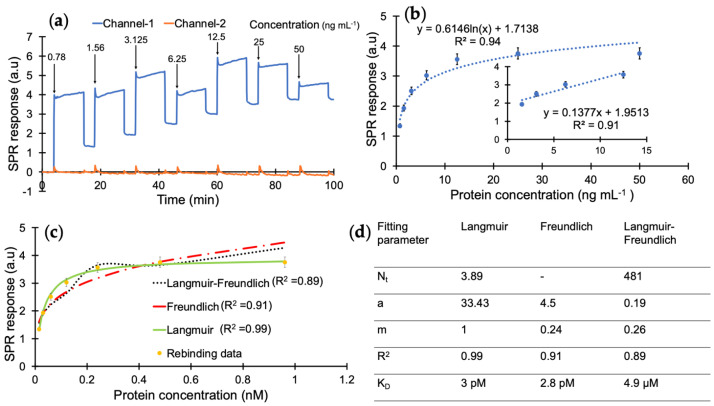
(**a**) Real-time SPR sensorgram of cumulative cTnI detection. (**b**) Concentration-dependent protein binding with a correlation coefficient of 0.94 and R^2^ value of 0.91 for the linear range of 1.56–12.5 ng mL^−1^. (**c**) Binding isotherm fittings for the interaction between the nanoMIPs and cTnI. (**d**) Binding isotherm fitting parameters obtained from Langmuir, Freundlich and Langmuir–Freundlich models.

According to the results of preliminary tests, the protein detection was further studied with a 10 min injection time in the concentration range of 0.78–50 ng mL^−1^ using a direct assay strategy (Figure 5a). The analyte samples prepared in PBS/T were injected onto the sensor surface from the least concentrated to the highest. The overall results of the concentration-dependent protein detection are provided in Figure 5b with the linear concentration range of 1.56–12.5 ng mL^−1^ with a correlation coefficient of 0.91 (Figure 5b, inset). 

A kinetic data analysis was performed to investigate the dissociation constant (K_D_) and determine the binding affinity between the cTnI and nanoMIP receptors. For this, the protein detection data in Figure 5b were subjected to Langmuir, Freundlich, and Langmuir–Freundlich isotherms to realize the binding behaviour of the receptor–analyte pair (Figure 5c). The K_D_ was found to be 2.99 × 10^−11^ M with the Langmuir model as the best fitting isotherm with an R^2^ value of 0.99 in the concentration range of 0.78–50 ng mL^−1^, which indicates a very high affinity of MIP receptors against their target protein (Figure 5d) and suggests one-to-one binding between the receptor and analyte [32,38]. On the other hand, the Langmuir–Freundlich and Freundlich models resulted in lower R^2^ values of 0.91 and 0.89, respectively. This further confirms that the imprinted cavities consist of homogenous binding sites. 

The developed portable SPR sensor performed better than the reported SPR systems for the detection of the cardiac troponin biomarkers in terms of affinity, sensitivity, specificity and handling simplicity (Table 1). For instance, several research works have been reported on the detection of cardiac biomarkers, which require heavy equipment to handle and special training to operate, in contrast to the portable and user-friendly SPR apparatus employed in this study. Of note, existing SPR sensors for cardiac troponin detection have been predominantly relying on antibody-based receptors and often requiring sandwich assays for obtaining desirable sensitivity (Table 1). These factors do not only increase the assay complexity but also the overall cost of the sensing platform. 

### 3.3. Electrochemical Characterization of NanoMIP Sensor

**Figure 6:**
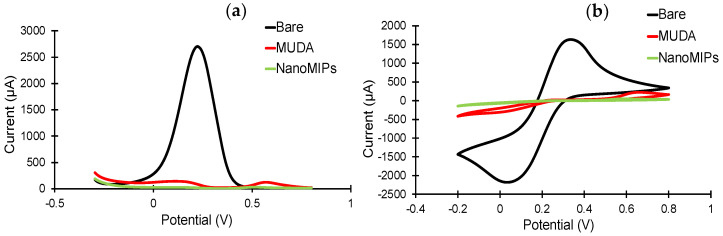
(**a**) Square-wave and (**b**) cyclic voltammograms confirm the immobilization of the nanoMIPs after SAM formation on the gold surface. All measurements were recorded using 10 mM K_3_(Fe(CN)_6_) in 0.1 M KCL at room temperature. All the represented voltammograms are the result of the average of three measurements.

The sensor fabrication steps were investigated in detail by employing electrochemical methods, including CV, SWV and EIS [45]. The sensor chip surface was characterized before and after MUDA coating, as well as after nanoMIP immobilization. As expected, the highest current signals in the CV and SWV measurements were demonstrated by the bare electrode (Figure 6), as the electrons in the redox marker solution were able to pass through the phase boundary with relative ease. For both CV and SWV, the current values significantly decreased after the formation of SAM compared to the bare electrode due to the hindered electron exchange at the electrode surface, indicating that the MUDA coating was successfully formed. Building a layer of nanoMIP impedes the flow of electrons, resulting in an almost complete signal suppression for both voltammetry curves [45]. SWV peaks demonstrate a peak suppression from 26,221.3 µA for bare to 134.24 and 24.7 µA for MUDA-coated and nanoMIP immobilized surfaces, respectively (Figure 6a). 

**Figure 7:**
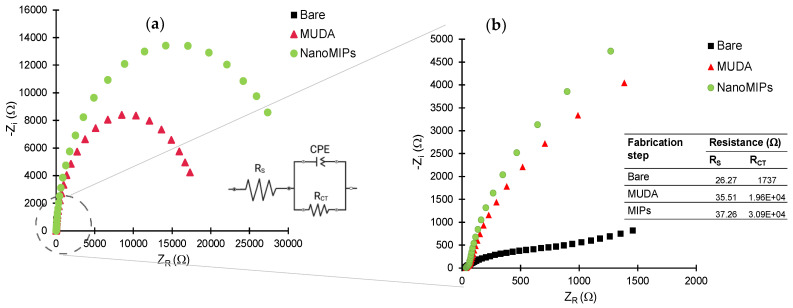
(**a**) Nyquist plots of the data obtained for bare gold electrode, MUDA and nanoMIP immobilization with inset equivalent circuit. (**b**) Zoomed Nyquist plots with inset table of resistivity of the solution (R_s_) and charge transfer resistance (R_CT_) of the circuit.

EIS measurements were also performed to further confirm all the fabrication steps prior to target rebinding studies. Figure 7 depicts the Nyquist plots for bare, MUDA-coated and nanoMIP immobilized surfaces. The inset (Figure 7a) represents the Randles equivalent circuit congruent to the EIS data consisting of the resistivity of the solution (R_s_), charge transfer resistance (R_CT_) and constant phase element (CPE) [46]. The value of R_CT_ (Figure 7b) increases with each fabrication step, symbolizing the successful deposition of MUDA and nanoMIP immobilization as the value of R_CT_ is inversely proportional to the current flowing through the surface. R_CT_ values correlate with the diameter of the semi-circle so, as the R_CT_ value increases, the diameter of the semi-circle increases. That is why the semi-circle part of the bare is insignificant as the R_CT_ value is very low due to the easy flow of charge through the bare surface.

### 3.4. AFM Characterization of NanoMIP Sensor 

The surface morphologies of the SPR sensor chip after each fabrication step were characterized using AFM. The surface topographies, the root-mean-square (RMS) roughness, phase images and 3D height images were investigated for bare surface, MUDA coated and nanoMIP immobilized surfaces, as well as after protein rebinding on the gold sensor chip in a 10 × 10 μm area. A nanoMIP solution was prepared to drop cast on the silicon wafers for the AFM measurements. 

**Figure 8:**
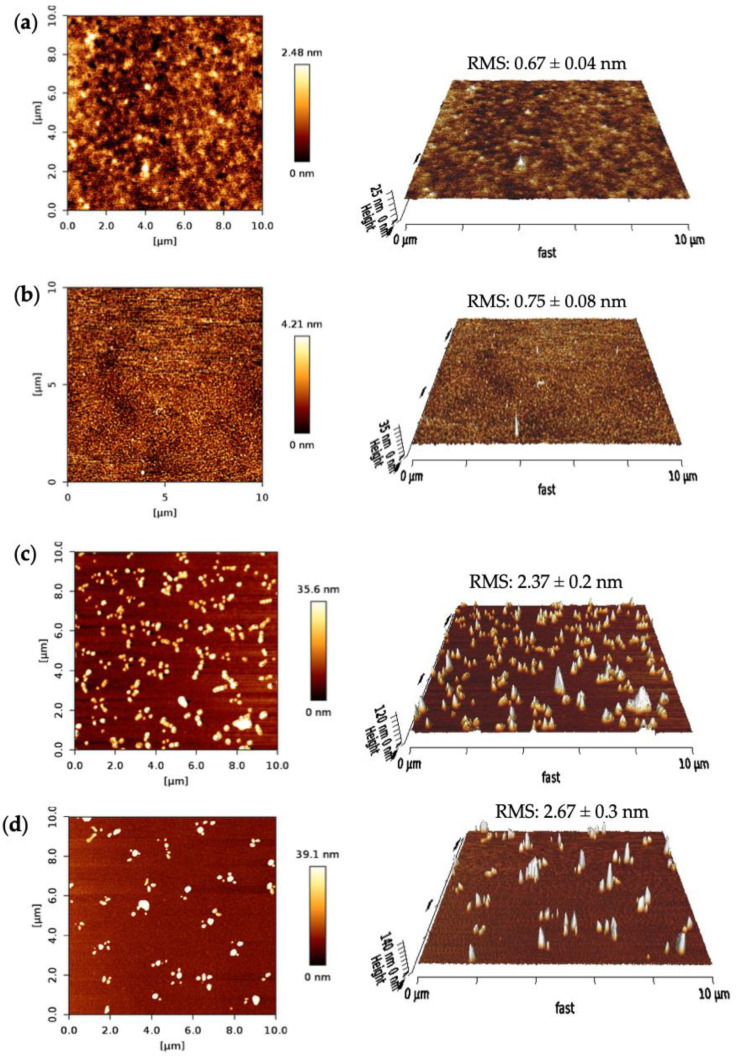
AFM 2D height images and 3D surface topographies of (**a**) bare, (**b**) MUDA coated, (**c**) nanoMIP immobilized and (**d**) protein-bound surfaces. All the RMS values are the result of 10 cross-sectional data.

Figure 8 depicts the AFM results of the fabrication steps for the nanoMIP sensor. The bare surface of the clean SPR chip had an RMS roughness value of 0.67 ± 0.04 nm with a uniform and smooth profile (Figure 8a). The RMS value had increased to 0.75 ± 0.08 nm and the 2D height to 4.21 nm after MUDA coating (Figure 8b). The surface got slightly rougher but still maintained its uniformity, confirming the successful formation of the MUDA layer. 

Further, the RMS value drastically increased upon nanoMIP immobilization to 2.37 ± 0.2 nm, indicating the successful immobilization of the molecular receptors. Moreover, after the protein rebinding, the surface got rougher (RMS = 2.67 ± 0.3 nm) and the 2D height increased by ~3.5 nm. Along with the RMS values, significant changes can be seen in the 2D images after every sensor fabrication step.

### 3.5. Selectivity Studies

**Figure 9:**
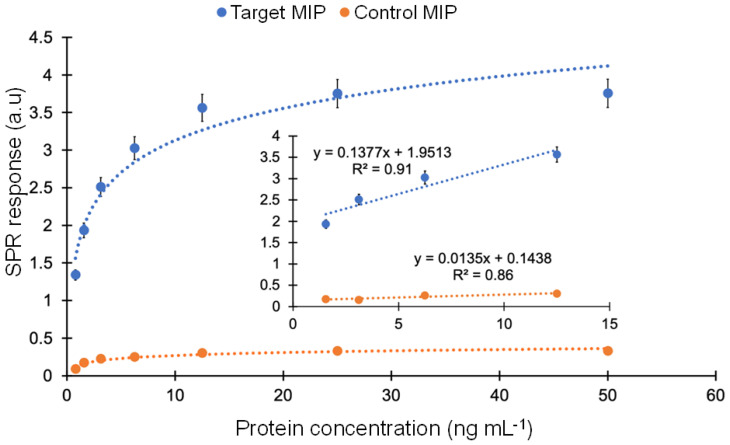
Comparison of affinity binding between the target and control MIPs in the concentration range of 0.78–50 ng mL^−1^.

By comparing the affinity with a control MIP, the selectivity of the MIP sensor towards cTnI was examined. To achieve this, cumulative protein rebinding tests were run using target and control nanoMIPs in the concentration range of 0.78–50 ng mL^−1^. A non-imprinted polymer (NIP) cannot be synthesized as solid phase synthesis requires templated solid support for polymerization. Therefore, a control nanoMIP was synthesized using the same procedure and a non-specific template (viral protein epitope). For the whole concentration range, the cTnI-specific nanoMIP exhibited a strong affinity toward cTnI. The average ratio between the responses of target and control MIPs in the concentration range of 0.78 to 50 ng mL^−1^ was determined to be 12.13, demonstrating very high selectivity of the target MIP toward cTnI (Figure 9).

### 3.6. Specificity Studies

**Figure 10:**
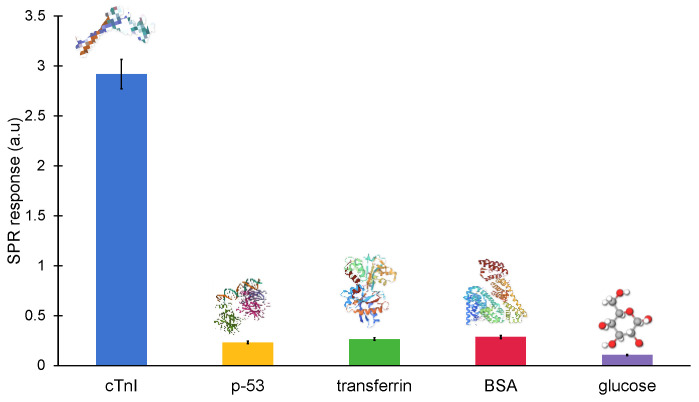
Cross-reactivity studies against non-specific molecules (p-53, transferrin, BSA, glucose) on the epitope-imprinted MIP sensor at a fixed concentration of 100 ng mL^−1^.

The majority of non-specific molecules, including antibodies, proteins, hormones, enzymes and mineral ions, are found in clinical samples. These naturally occurring biomolecules could potentially interact with the MIP cavities and interfere with the signal in an unwanted way. As a result, a sensor’s specificity is essential for the precise detection of the target biomarker. Hence, the binding of cTnI-specific nanoMIPs was also tested with non-specific molecules, such as glucose, BSA, transferrin and p53, at a constant concentration of 100 ng mL^−1^ in order to determine the specificity of the binding between the cTnI protein and its particular nanoMIPs (Figure 10). The non-specific binding of the reference molecules was identified as 8.034% (p-53), 9.153% (transferrin), 3.656% (glucose) and 9.85% (BSA), demonstrating a high specificity against cTnI. 

## 4. Conclusions

In this work, a biomimetic sensor was developed for the detection of cTnI protein using a portable angular SPR and epitope-imprinted synthetic receptors. The novel SPR technique offers unique advantages such as being rapid, cost-efficient, real-time, user-friendly and label-free. Moreover, it also does not require complex assay strategies, such as sandwich or competitive assays, for obtaining high sensitivity. The portable nanoMIP-SPR sensor has shown good reproducibility and sensitivity for cTnI quantification in the concentration range of 0.78–50 ng mL^−1^ with a calculated LOD of 0.52 ng mL^−1^. The dissociation constant was found to be 2.99 × 10^−11^ M using a Langmuir fitting model, revealing an excellent affinity of nanoMIPs towards the cTnI biomarker. The sensor fabrication steps were successfully confirmed using AFM as well as electrochemical methods (i.e., SWV, CV and EIS). The selectivity studies with control nanoMIPs proved that the target nanoMIPs exhibit 12-fold higher binding affinity toward cTnI protein. Moreover, the sensor specificity was also determined by studying with reference molecules, where the non-specific binding of interferants was found to be in the range of 3–10%. Hence, the developed biomimetic SPR sensor has great potential to be further enhanced for point-of-care testing and the early diagnosis of AMI. 

## Figures and Tables

**Table 1:** The list of various SPR biosensors used for the detection of cardiac biomarkers reported in the literature and the current work.

Sensor Type	Biomarker	Receptor Type	Assay Type	Concentration Range (ng mL^−1^)	LOD (ng mL^−1^)	K_D_ (M)	Ref
Portable SPR	cTnI	NanoMIPs	Direct	0.78–50	0.52	2.99 × 10^−11^	This work
Biacore SPR	cTnT	Antibody	Sand-wich	5–400	5	3.28 × 10^−9^	[39]
Fiber optic SPR	cTnI	Antibody	Direct	2500–4000	1.4	-	[11]
Auto lab spirit	cTnT	Antibody	Direct	0.03–6.5	0.01	-	[40]
Navi 200 SPR	cTnT	Antibody	Direct	50–100	100	-	[41]
SPF	cTnI	Antibody	Direct	3.9–100	0.98	-	[42]
SPR	cTnI	Antibody	Sand-wich	0.5–20	0.25	-	[43]
SPR	cTnT	Antibody	Sand-wich	5–300	3.6	-	[44]

SPF: Surface plasmon-enhanced fluorescence.

## Data Availability

The data presented in this study are available on request from the corresponding author.
